# Identification of Non-Electrophilic Nrf2 Activators from Approved Drugs

**DOI:** 10.3390/molecules22060883

**Published:** 2017-05-26

**Authors:** Qing-Ye Zhang, Xin-Yi Chu, Ling-Han Jiang, Meng-Yuan Liu, Zhi-Ling Mei, Hong-Yu Zhang

**Affiliations:** 1 Hubei Key Laboratory of Agricultural Bioinformatics, College of Informatics, Huazhong Agricultural University, Wuhan 430070, China; chuxy@webmail.hzau.edu.cn (X.-Y.C.); jianglinghan@hotmail.com (L.-H.J.); liumengyuan2017@outlook.com (M.-Y.L.); 2 Shanghai Thinkgene Biotech Co., LTD., Shanghai 200000, China; thinkgene@yeah.net

**Keywords:** oxidative stress, Nrf2 activator, drug repositioning, redox regulators

## Abstract

Oxidative damage can lead to a wide range of diseases. Nrf2 is an important transcription factor that regulates many of the cytoprotective enzymes involved in the oxidative stress response. Therefore, targeting the regulation of Nrf2 activation is one logical and effective strategy to prevent or lower the risk of oxidative stress-related diseases. Until now, most research has focused on electrophilic indirect Nrf2 activators, but the risk of ‘off-target’ effects may be associated with these activators. To find novel small non-electrophilic modulators of Nrf2, we started from chemical agents derived from a connectivity map (cMap) and identified 22 non-electrophilic potential Nrf2-activating drugs through a drug repositioning tactic. By determining the expression changes of antioxidant genes in MCF7 cells that were treated with the potential Nrf2 activators using quantitative real-time polymerase chain reaction RT-PCR (real-time polymerase chain reaction) (qRT-PCR), astemizole was found to have a greater scale of upregulating antioxidant genes *NQO1*, *HO-1*, and *GCLM* than the positive control d,l-sulforaphane, although the testing concentration was lower than that of the control. Astemizole is a good potential redox regulator and deserves more pharmacodynamic experimentation to test and verify its feasibility for use as an Nrf2 activator.

## 1. Introduction

Developing effective means to protect against metabolic oxidative stress and exposure to environmental toxicants is an essential requirement for the evolution of early eukaryotic life [1]. Oxidative stress is aroused by the imbalance in reactive species and the antioxidative defense systems in living cells, which can damage all cellular macromolecules, including proteins, lipids, and DNA [2]. This oxidative damage could be a common mechanism for a wide range of disorders, which include chronic inflammation, cancers, cardiovascular diseases, neurodegenerative diseases, and aging [3]. To counteract oxidative stress, cells switch on the antioxidative defense system to protect against and recover from the damage to the cell by upregulating a battery of cytoprotective genes, including antioxidant and detoxifying enzymes [4].

Nrf2 (nuclear factor (erythroid-derived 2)-like 2) is an important transcription factor that regulates many of the cytoprotective enzymes that are involved in the adaptive oxidative stress response [5]. Nrf2 is a member of the cap ‘n’ collar (CNC) subfamily of basic region leucine zipper (bZIP) transcription factors. Kelch-like ECH (chicken homologous to Nrf2)-associated protein 1 (Keap1) is used as a stress sensor of Nrf2. Under unstressed conditions, Keap1 binds to Nrf2, which promotes the ubiquitination and subsequent proteolysis of Nrf2. This mechanism maintains a low level of Nrf2 protein in the cell. Upon activation in response to oxidative and electrophilic stress, Nrf2 detaches from Keap1, and the degradation is stopped. Nrf2 rapidly accumulates, migrates to the nucleus, forms heterodimers with small musculoaponeurotic fibrosarcoma (Maf) proteins, and binds to the antioxidant response element (ARE) sequence to activate the transcription of a wide array of multiple antioxidant, detoxifying, and cell survival genes against oxidative and xenobiotic stress [6,7,8]. The Keap1-Nrf2-ARE pathway can activate ARE-dependent genes, encoding a group of approximately 500 proteins that are expressed in various isoforms and distributed in various organelles and subcellular compartments. These antioxidant and detoxifying enzymes such as NAD(P)H quinone oxidoreductase 1 (NQO1), heme oxygenase 1 (HO1), and glutamate-cysteine ligase (GCL), which consists of a catalytic subunit (GCLC) and a modulatory subunit (GCLM), are upregulated by Nrf2 and have important roles in protecting cells against oxidative stress; thus they serve as common biomarkers for evaluating the Nrf2 dependent transcription [3,9,10].

Based on the comprehensive and critical models of Nrf2-regulated genes, the Keap1-Nrf2-ARE pathway has served as a high-value therapeutic target for diseases and conditions involving oxidative stress [11]. Targeting the regulation of the activation of Nrf2 could be one logical and effective strategy to prevent and lower the risk of a number of diseases [12]. The protective roles of Nrf2 activation in cancers [13,14,15,16], Alzheimer’s disease [17], Parkinson’s disease [18], Huntington’s disease [19,20], and amyotrophic lateral sclerosis [21,22] have been well established. To date, the activation of Nrf2 can take place through either a direct antioxidant or indirect antioxidant mechanism, and most of the research has focused on electrophilic indirect Nrf2 activators [3,23,24]. Up until now, indirect activators have been classified into several classes based on their chemical structures and modes of interaction with cysteine sulfhydryl groups: (1) oxidizable phenols and quinones; (2) dithiolethiones; (3) isothiocyanates and sulfoxythiocarbamates; (4) trivalent arsenicals; (5) vicinal dimercaptans and diallyl sulfides; (6) Michael acceptors; (7) polyenes; (8) heavy metals and metal complexes; (9) hydroperoxides; and (10) selenium-based compounds [3,10,25,26,27,28,29,30,31].

Although several successful electrophilic indirect Nrf2 activators have been approved and used as first-line oral drugs, the risk of ‘off-target’ effects may be associated with them due to the complex molecular mechanisms of action of the traditional electrophilic Nrf2 activators, which covalently bind to the thiol of the cysteine in Keap1. This may lead to unwanted binding between the activators and cysteine-containing proteins [32,33]. Recently, novel small direct or non-electrophilic modulators of the Keap1-Nrf2-ARE pathway have been discovered [4,34,35,36,37,38,39,40,41,42], but it is still urgent to develop more small-molecule antioxidants as therapies for many diseases [43,44].

De novo drug discovery is a very expensive and time-consuming process. Therefore, drug repositioning has played a critical role in the modern pharmaceutical industry. The gene expression data, in particular those contained in the connectivity map (cMap), have been widely used in drug repurposing and lead discovery [45,46]. cMap is a database comprising gene expression profiles for human cell lines treated with 1309 agents. A recent biclustering analysis of cMap data generated a total of 49 modules for gene expression profiles, and module 36 is regulated by Nrf2 [47]. This module was linked with 189 chemical agents, which were considered potential redox regulators through activating Nrf2 [47]. This provides us an with opportunity to quickly and effectively find novel small non-electrophilic modulators of Nrf2. In this study, all the agents coupled with module 36 were analyzed and screened based on their chemical characteristics and approved information for further biological testing.

## 2. Results and Discussion

### 2.1. Screening of Potential Redox Regulators

According to our previous study [47], there are 189 agents attached to the Nrf2 transcription factor. Some of them are known antioxidants such as quercetin, butein, nordihydroguaiaretic acid, myricetin, kaempferol, genistein, ascorbic acid, and ebselen. Moreover, four para-quinones (i.e., tanespimycin, 1,4-chrysenequinone, menadione, and tetroquinone) are also included in this list, which abstract electrons and protons from the thiols of Keap1 and result in the activation of Nrf2 [48]. Therefore, the 189 agents coupled with Nrf2 are likely potential redox regulators. 

To reduce the time and money consumption of the drug discovery process, drug repositioning is an important method. In this study, 86 approved drugs were screened out from the 189 agents for further research (Table S1). To find novel small non-electrophilic modulators of Nrf2, all the agents with an electrophilic substructure were deleted from the approved drug list. Finally, 22 approved non-electrophilic drug compounds were selected and divided into seven categories according to their structure characteristics (Table 1). The seven categories are: phenothiazine (type-I), tricyclic antidepressants (type-II), trihexyphenidyl (type-III), phenyl pyridine (type-IV), quinolin-8-substitute (type-V), tamoxifen (type-VI), and hexetidine (type-VII).

### 2.2. Potency of Candidate Compounds to Activate the Expression of Nrf2-Regulated Cytoprotective Genes

To further inspect the redox regulator candidates’ potency to activate Nrf2, one or two representatives from each category were selected to carry on experimental validation (as shown in Table 2). In order to ensure that the selected structures are representative, the compounds with common forms and large changes in substituents were selected. The time-dependent increases in the expression levels of Nrf2-regulated genes after treatment with known redox regulators and candidates at various time points were sampled and analyzed. In this study, the known redox regulator d,l-sulforaphane served as a positive control, and DMSO or chloroform solvent was used as the negative control. We measured the expression changes of antioxidant genes *NQO1*, *HO-1*, and *GCLM* in cells treated with nine different candidate drugs using quantitative RT-PCR (qRT-PCR). These three genes are well-characterized transcriptional targets of Nrf2 and are widely used in the evaluation of the effect of the Nrf2 regulators [3,9,10]. The expression levels of these genes at 6 h, 12 h, and 24 h after treatment with the nine candidates were measured. The concentrations of the candidates were determined based on the reported information (as shown in Table 2).

**Figure 1:**
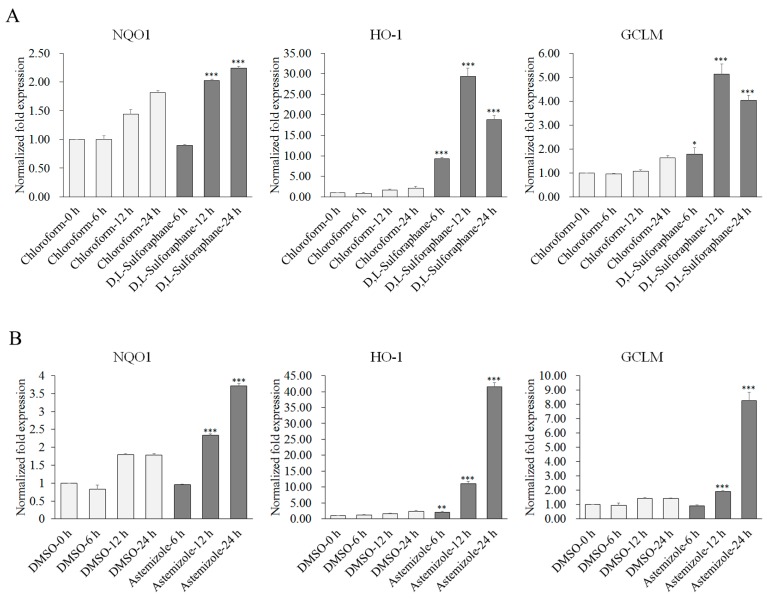

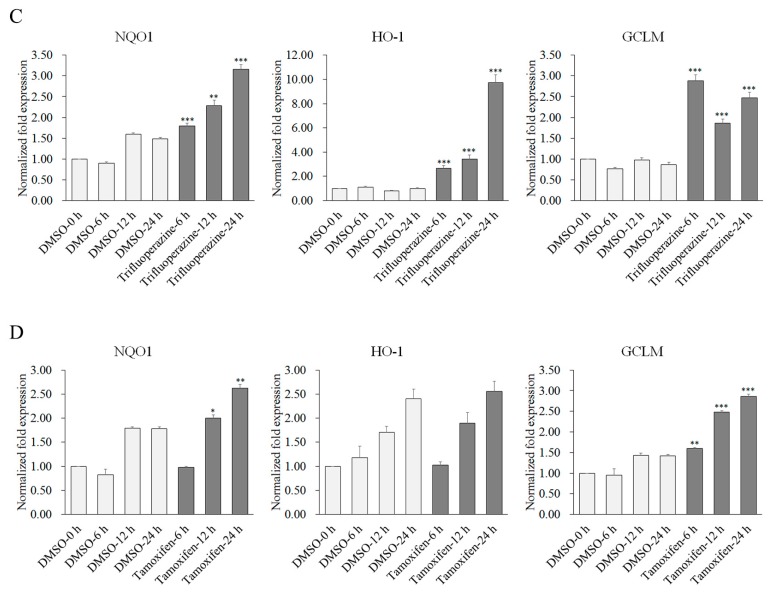
The expressions of *NQO1*, *HO-1*, and *GCLM* genes after treatment with the positive control and the potential Nrf2-activating drugs at 6 h, 12 h, and 24 h time points. (**A**) The results of samples treated with the positive control d,l-sulforaphane (15 μM). (**B**), (**C**), and (**D**) The results of samples treated with astemizole (8 μM), trifluoperazine (10 μM), and tamoxifen (1 μM), respectively. The genes’ expression levels at 0 h are normalized to 1. Bars represent the average standard deviations, *n* = 3. The significance of the expression fold changes between samples treated with drugs and negative control at the same time points are tested using a paired *t*-test: * *p* < 0.05; ** *p* < 0.01; *** *p* < 0.001.

All the experimental results are shown in histograms displayed in Figure 1 and Figure S1 to Figure S6. The expression levels of *NQO1*, *HO-1*, and *GCLM* genes were determined using β-actin as a loading control. The experimental results showed that, compared to the negative control, almost all of the nine candidates displayed the function of upregulating antioxidant genes *NQO1*, *HO-1*, and *GCLM*; six of the nine candidates displayed the function of upregulating all of the three genes. Most of the results showed that the expression levels of the three genes increased gradually with the time change. These results preliminarily validated the effectiveness of our drug repositioning tactic.

**Figure 2:**
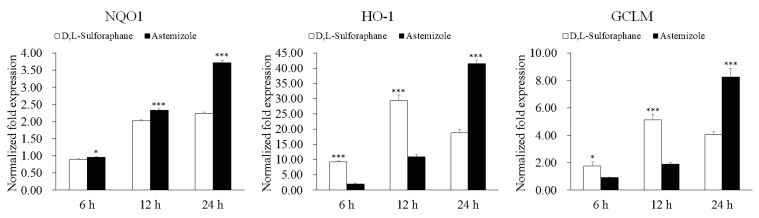
The comparison of NQO1, HO-1, and GCLM expression changes after treatment with d,l-sulforaphane (15 μM) and astemizole (8 μM) at 6 h, 12 h, and 24 h time points. Bars represent the average standard deviations, *n* = 3. The significance of the expression fold changes between samples treated with d,l-sulforaphane and astemizole at the same time points are tested using a paired *t*-test: * *p* < 0.05; *** *p* < 0.001.

Among these candidates, astemizole showed the strongest ability to upregulate the antioxidant genes *NQO1*, *HO-1*, and *GCLM*. The scale of the upregulation of the three genes in the cells incubated with astemizole was even larger than that in the cells incubated with d,l-sulforaphane at the 24 h time point, although the testing concentration of astemizole was nearly less than one half of that of d,l-sulforaphane (Figure 2). Further analysis detected that there were similar trends in the upregulation of the antioxidant genes *NQO1*, *HO-1*, and *GCLM* between astemizole and d,l-sulforaphane (Figure 1A,B). They both have the greatest impact on the *HO-1* gene and then on the *GCLM* gene, with the smallest impact being found on the *NQO1* gene. From the perspective of the intensity of action with time, d,l-sulforaphane reached a peak in upregulating the antioxidant genes *GCLM* and *HO-1* at 12 h, and astemizole has a significant upregulation of these two genes between 12 h and 24 h. Thus, it may be still not reach its peak at 24 h. The ability of astemizole to upregulate the antioxidant genes has also been observed in a rat module. Lee and colleagues found that oxidative stress response related genes are upregulated in the cardiac tissue and blood mononuclear cells of astemizole treated rats [51].

Astemizole is a second-generation antihistamine drug, which acts as a histamine H1-receptor antagonist and suppresses the formation of edema caused by histamine [52]. Previous researches prevent that edema may result from reactive oxygen species (ROS) and could be relieved by some antioxidants [53]. This suggests that astemizole may serve as an antioxidant. The regulation of ion channels by ROS has been suggested to be associated with some pathological conditions, including liver diseases [54]. Astemizole is also a nonspecific inhibitor of Kv10.1 and Kv11.1 potassium channels, and it could significantly decrease cell proliferation, increase apoptosis, and clearly prevent hepatocellular carcinoma (HCC) development in vivo [55]. HCC represents 80% of primary liver cancers, which are mainly caused by chronic inflammation, with severe oxidative stress leading to fibrosis and then cirrhotic livers [56,57]. Our existing results showed that astemizole is a good potential redox regulator, which could regulate oxidative stress by activating Nrf2. The results may lend evidence to prove that it could be able to regulate histamine receptors and ion channels to achieve the regulation of oxidative stress. Astemizole deserves more pharmacodynamic experimentation to test and verify its feasibility for use as an Nrf2 activator.

Although the abilities of the candidates to upregulate the antioxidant gene *GCLM* are all lower than that of d,l-sulforaphane ,except for astemizole, several drugs show stronger abilities to upregulate the *NQO1* gene than d,l-sulforaphane, such as trifluoperazine (Figure 1C), tamoxifen (Figure 1D), and diphenylpyraline (Figure S4). Moreover, the expression of the *HO-1* gene has a larger increase after treatment with trifluoperazine (10 μM) at the 24 h time point, while d,l-sulforaphane has begun to decrease at that time (Figure 1A).

Trifluoperazine is a phenothiazine, and its primary application is for schizophrenia [58]. It has also been described as modifying P-type ATPase activity [59], inhibiting HIV-associated apoptosis [60], and preventing ROS damage by Fe/H2O2 [61]. Trifluoperazine was proven to be able to decrease the intracellular ROS accumulation and alleviate oxidative damage to cells [62]. Therefore, the present finding provides evidence to support the opinion that trifluoperazine may be useful in preventing cell damage in some situations where oxidative stress is expected to take place [62,63].

Tamoxifen is an approved anticancer drug to prevent breast cancer by inducing oxidative damage. However, recently, several clinical resistances to tamoxifen were reported, and the resistance mechanism was demonstrated to be that the breast cancer cells could increase the expression of Nrf2 and the subsequently activated *ARE* genes could respond to the tamoxifen-induced oxidation [64,65]. Although tamoxifen has the smallest concentration of 1 μM, it still showed the potency to upregulate *NQO1*, *HO-1*, and *GCLM* genes in our experiment (Figure 1D). This is consistent with the reported results and also suggests that reducing the tamoxifen-induced activation of Nrf2 may be a key factor to overcome the clinical resistance to the drug.

## 3. Materials and Methods

### 3.1. Data and Reagent Retrieval

The 189 potential redox regulators coupled with Nrf2 were downloaded from the reference [47]. All the tested compounds were purchased from Sigma-Aldrich (St. Louis, MO, USA). The MCF7 cells were purchased from the Cell Bank of the Chinese Academy of Science (http://www.cellbank.org.cn/detail_1.asp?id=152&serial=TCHu%2074).

### 3.2. Cell Culture Conditions 

The MCF7 cells were cultured in minimum essential medium (MEM) (GIBCO, 41500034, Thermo Fisher Scientific Inc., Waltham, MA, USA) supplemented with 10% fetal bovine serum (FBS) in a humidified atmosphere of 5% CO_2_ and 95% air at 37 °C. Then, the MCF7 cells were seeded in 12-well plates (Corning, 3513) and exposed to different concentrations of test compounds separately. d,l-sulforaphane served as a positive control, while DMSO (dimethyl sulfoxide) or chloroform served as the negative control. After incubation for 6 h, 12 h, or 24 h, the medium was removed and the cells were harvested for further analysis.

### 3.3. RNA Extraction and qRT-PCR Analysis

The total RNA of the MCF7 cells was isolated using an RNeasy Mini kit (Qiagen, 74104, Hilden, North Rhine-Westphalia, Germany). The quantity and purity of RNA samples were assessed by the a NanoDrop One Microvolume UV-Vis Spectrophotometer (Thermo Scientific, Thermo Fisher Scientific Inc., Waltham, MA, USA), and RNA samples with an A260/A280 ratio of 1.9–2.2 were stored at −80 °C for further analysis. The RNA was reverse transcribed by the TOYOBO (FSQ-101, Kita, Osaka, Japan) reagent kit following the manufacturer’s instructions.

Quantitative real-time RT-PCR analyses of NQO1, HO-1, and GCLM were performed on the LightCycler@96 Real Time PCR system (Roche, Aigle, Vaud, Switzerland) using iTaq Universal SYBR Green Suoermix (Bio-Rad, Hercules, CA, USA). The primers used for qRT-PCR are listed in Table 1. β-actin was used for normalization. The PCR conditions were as follows: 95 °C for 1 min; 40 cycles of degeneration at 95 °C for 10 s, annealing at 57 °C for 10 s, and extension at 72 °C for 15 s; then degeneration at 95 °C for 1 min and annealing at 55 °C for 1 min. Finally, the dissociation curves were generated by increasing the temperature from 65 to 95 °C stepwise by 0.5 °C every 10 s. All the qPCR analyses were performed in triplicate.

## 4. Conclusions

In this study, 86 approved drugs were screened from the 189 potential Nrf2-related agents derived from cMap data. Among these compounds, 22 non-electrophilic drugs were selected and divided into seven categories for further analysis. Nine candidate compounds from the seven categories were selected to validate their potencies to activate Nrf2. The expression changes of antioxidant genes *NQO1*, *HO-1*, and *GCLM* in MCF7 cells treated with the nine candidate drugs were analyzed at various time points using qRT-PCR. The experimental results showed that almost all of the candidates indeed displayed the ability to upregulate the expressions of the antioxidant genes to a certain extent, and most of the expression quantities increase gradually with time. Astemizole was found to have a greater scale upregulation of the antioxidant genes than the known redox regulator d,l-sulforaphane at a lower testing concentration than that of the control. In addition, several drugs upregulate the *NQO1* gene to a greater extent than d,l-sulforaphane, such as trifluoperazine, diphenylpyraline, and tamoxifen. Moreover, the expression of the *HO-1* gene has a larger increase after treatment with trifluoperazine (10 μM) at the 24 h time point. The above results show the application value of the drug repositioning tactic in novel non-electrophilic modulators of Nrf2.

## Figures and Tables

**Table 1:** The seven selected categories of non-electrophilic redox regulators candidates.

**Type-I**	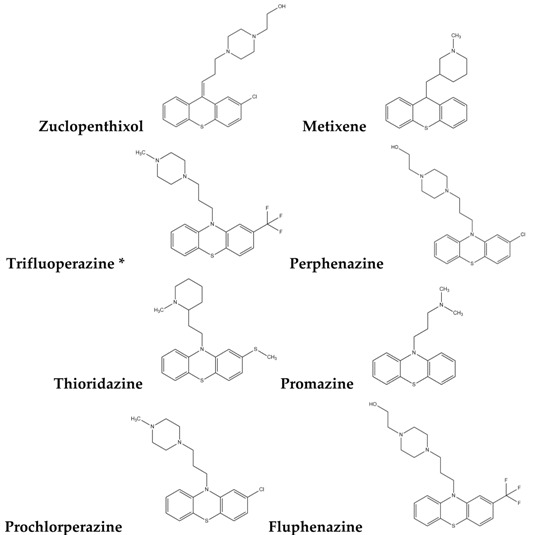
Type-II	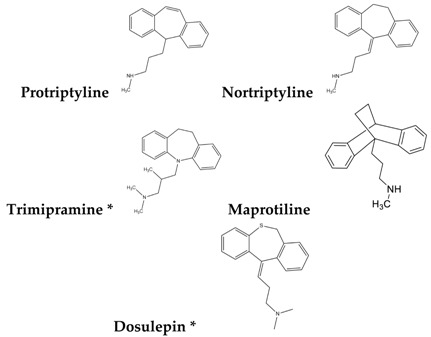
Type-III	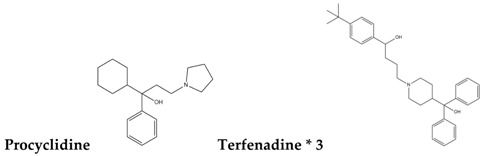
Type-IV	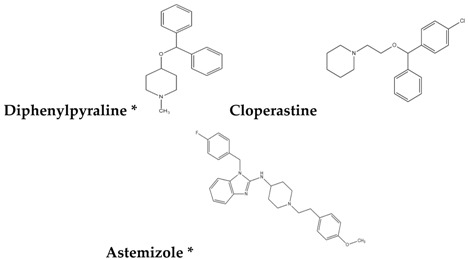
Type-V	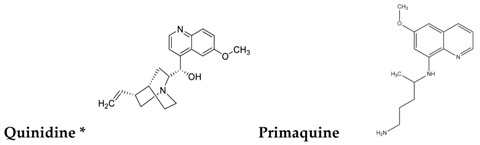
Type-VI	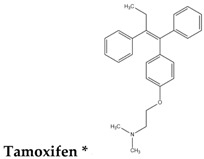
Type-VII	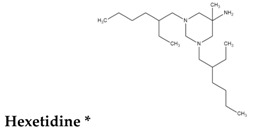

* Selected for further experimental evaluation.

**Table 2:** The information about the tested samples.

Drug	Solvent	Concentration	Sample
Terfenadine	Chloroform	8 μM [49]	6 h/12 h/24 h
Trimipramine	Chloroform	10 μM [49]	6 h/12 h/24 h
Quinidine	Chloroform	10 μM [49]	6 h/12 h/24 h
Hexetidine	Chloroform	10 μM [49]	6 h/12 h/24 h
Dosulepin	Chloroform	12 μM [49]	6 h/12 h/24 h
Diphenylpyraline	Chloroform	12 μM [49]	6 h/12 h/24 h
d,l-Sulforaphane	Chloroform	15 μM [50]	6 h/12 h/24 h
Tamoxifen	DMSO	1 μM [49]	6 h/12 h/24 h
Astemizole	DMSO	8 μM [49]	6 h/12 h/24 h
Trifluoperazine	DMSO	10 μM [49]	6 h/12 h/24 h
Chloroform (control)			0 h/6 h/12 h/24 h
DMSO (control)			0 h/6 h/12 h/24 h

All of the data are obtained from MCF7 cell line.

## Supplementary Materials

Supplementary materials are available online.

## Abbreviations

cMap	connectivity map
qRT-PCR	quantitative real time polymerase chain reaction
ARE	antioxidant response element
MEM	minimum essential medium
FBS	fetal bovine serum
DMSO	dimethyl sulfoxide
ROS	reactive oxygen species
