# Supplementary material for: Identification of Non-Electrophilic Nrf2 Activators from Approved Drugs
Source: Molecules. 2017 May 26;22(6):883. doi: 10.3390/molecules22060883 (PMC6152778; doi:10.3390/molecules22060883)
Supplement: Supplementary file 1 [file molecules-22-00883-s001.zip › supplementary_figures.docx]

**Supplementary figures**


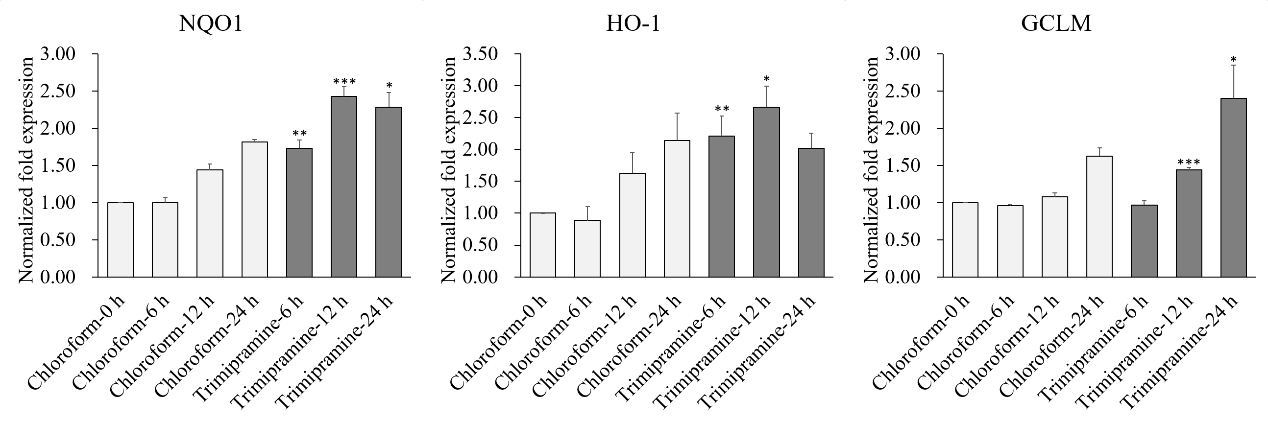


**Figure S1.** The comparison of NQO1, HO-1 and GCLM expression changes after treatment with trimipramine (10 μM) at 6 h, 12 h and 24 h time points. Bars represent the average standard deviations, n = 3. The significance of the expression fold changes between samples treated with trimipramine and negative control at the same time points are tested using paired t-test: *p < 0.05; **p < 0.01; ***p < 0.001.


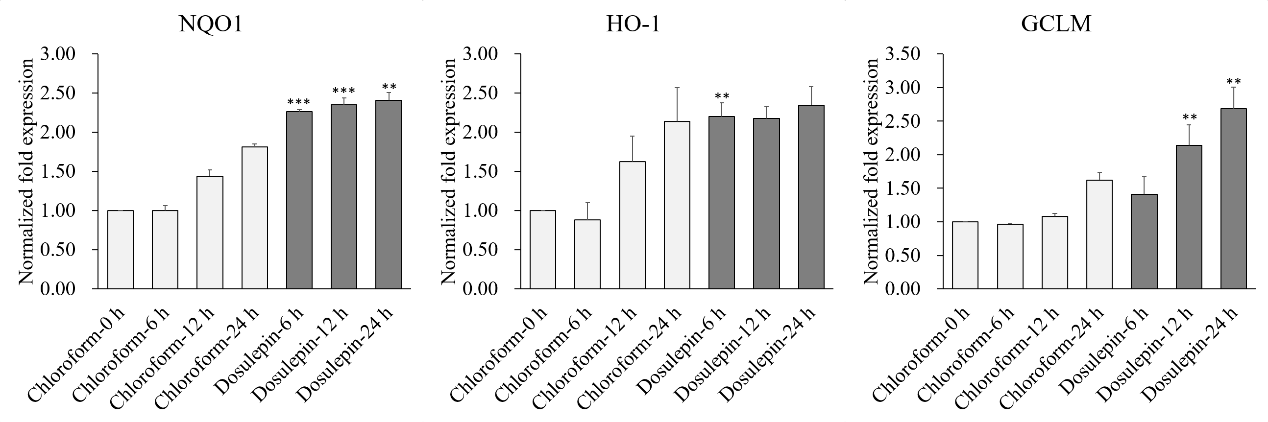


**Figure S2.** The comparison of NQO1, HO-1 and GCLM expression changes after treatment with dosulepin (12 μM) at 6 h, 12 h and 24 h time points. Bars represent the average standard deviations, n = 3. The significance of the expression fold changes between samples treated with dosulepin and negative control at the same time points are tested using paired t-test: *p < 0.05; **p < 0.01; ***p < 0.001.


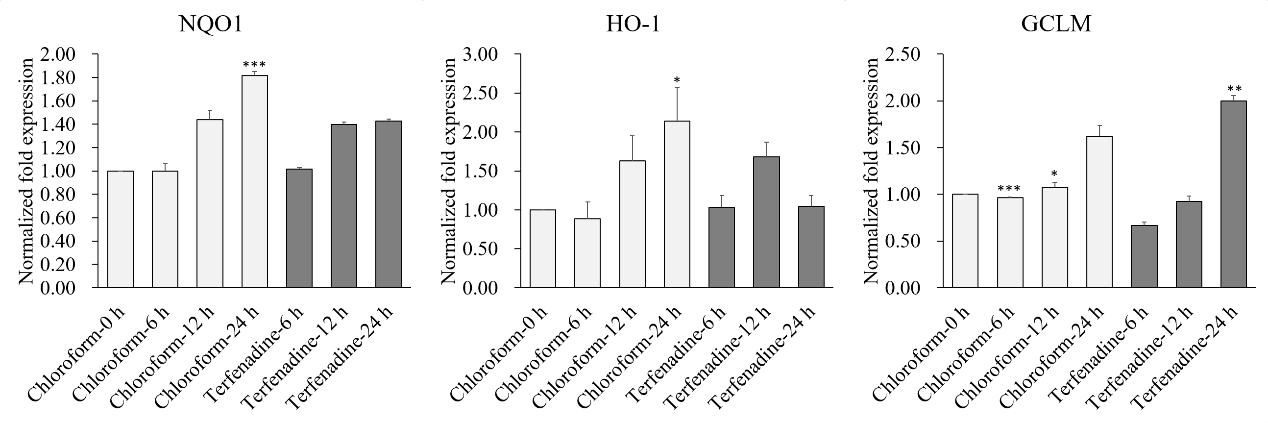


**Figure S3.** The comparison of NQO1, HO-1 and GCLM expression changes after treatment with terfenadine (8 μM) at 6 h, 12 h and 24 h time points. Bars represent the average standard deviations, n = 3. The significance of the expression fold changes between samples treated with terfenadine and negative control at the same time points are tested using paired t-test: *p < 0.05; **p < 0.01; ***p < 0.001.


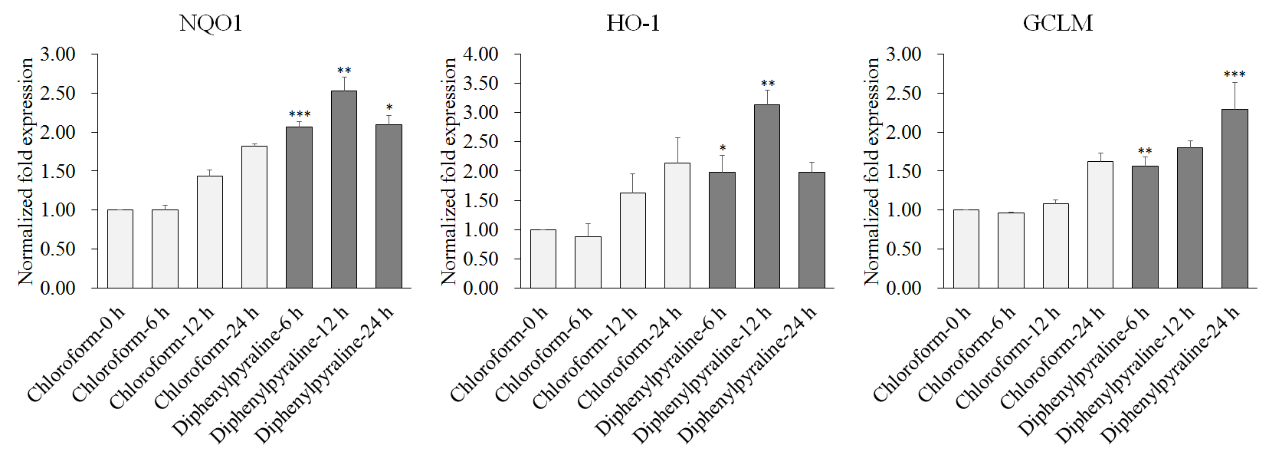


**Figure S4.** The comparison of NQO1, HO-1 and GCLM expression changes after treatment with diphenylpyraline (12 μM) at 6 h, 12 h and 24 h time points. Bars represent the average standard deviations, n = 3. The significance of the expression fold changes between samples treated with diphenylpyraline and negative control at the same time points are tested using paired t-test: *p < 0.05; **p < 0.01; ***p < 0.001.


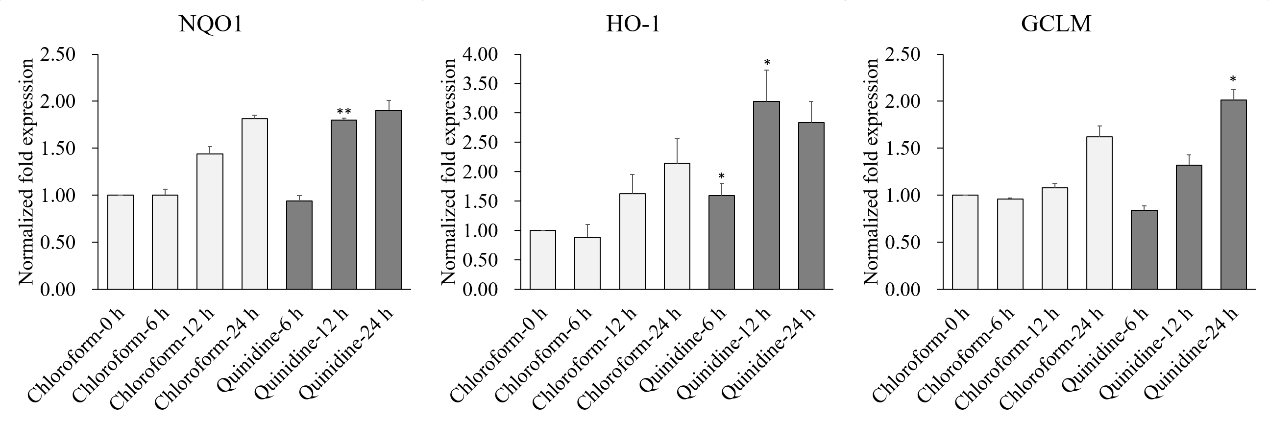


**Figure S5.** The comparison of NQO1, HO-1 and GCLM expression changes after treatment with quinidine (10 μM) at 6 h, 12 h and 24 h time points. Bars represent the average standard deviations, n = 3. The significance of the expression fold changes between samples treated with quinidine and negative control at the same time points are tested using paired t-test: *p < 0.05; **p < 0.01; ***p < 0.001.


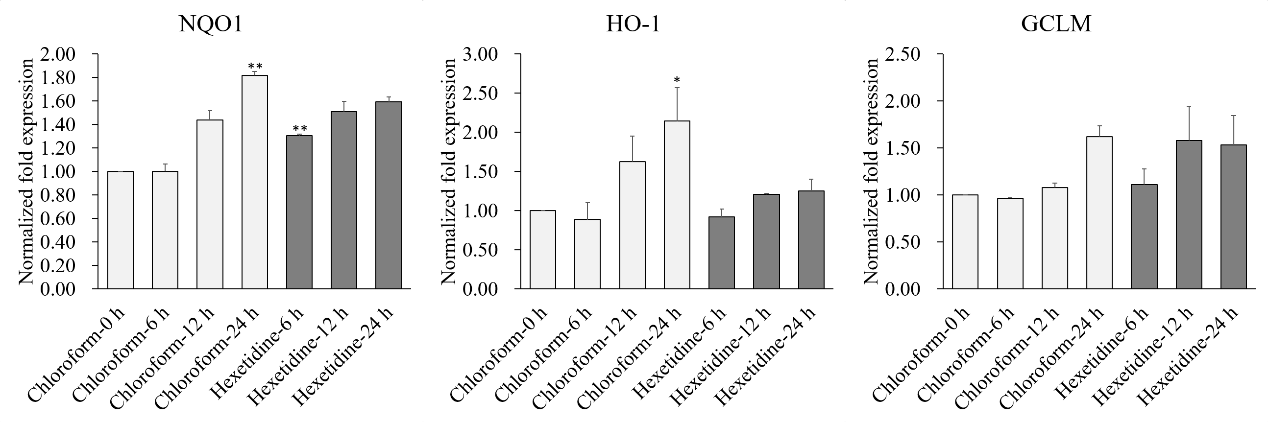


**Figure S6.** The comparison of NQO1, HO-1 and GCLM expression changes after treatment with hexetidine (10 μM) at 6 h, 12 h and 24 h time points. Bars represent the average standard deviations, n = 3. The significance of the expression fold changes between samples treated with hexetidine and negative control at the same time points are tested using paired t-test: *p < 0.05; **p < 0.01; ***p < 0.001.
