# Supplementary material for: Identification of Non-Electrophilic Nrf2 Activators from Approved Drugs
Source: Molecules. 2017 May 26;22(6):883. doi: 10.3390/molecules22060883 (PMC6152778; doi:10.3390/molecules22060883)
Supplement: Supplementary file 1 [file molecules-22-00883-s001.zip › Supplementary_Table_S1.docx]

**Supplementary Table S1.** The screened out 86 approved drugs.

| **Drug source** | **Drug source id** | **Drug name** |
| --- | --- | --- |
| Drug Bank | DB01118 | Amiodarone |
| Drug Bank | DB00681 | Amphotericin B |
| ClinicalTrials | NCT01322061 | Ascorbic Acid |
| Drug Bank | DB00637 | Astemizole |
| Drug Bank | DB00993 | Azathioprine |
| Drug Bank | DB01602 | Bacampicillin |
| Drug Bank | DB00389 | Carbimazole |
| Drug Bank | DB00262 | Carmustine |
| Drug Bank | DB00291 | Chlorambucil |
| Drug Bank | DB00527 | Cinchocaine |
| Drug Bank | DB09002 | Cloperastine |
| Drug Bank | DB01147 | Cloxacillin |
| Drug Bank | DB09028 | Cytisine |
| Drug Bank | DB00255 | Diethylstilbestrol |
| Drug Bank | DB08792 | Diloxanide |
| Drug Bank | DB01146 | Diphenylpyraline |
| Drug Bank | DB00822 | Disulfiram |
| Drug Bank | DB09167 | Dosulepin |
| Drug Bank | DB00645 | Dyclonine |
| Drug Bank | DB00378 | Dydrogesterone |
| Therapeutic Targets Database | DAP001372 | Ebselen |
| Drug Bank | DB02187 | Equilin |
| Drug Bank | DB00773 | Etoposide |
| Drug Bank | DB01216 | Finasteride |
| Drug Bank | DB00324 | Fluorometholone |
| ClinicalTrials | NCT02473250 | Fluoxetine |
| Drug Bank | DB00875 | Flupentixol |
| Drug Bank | DB00623 | Fluphenazine |
| ClinicalTrials | NCT00705341 | Fluticasone |
| ClinicalTrials | NCT02447328 | Fulvestrant |
| Drug Bank | DB00614 | Furazolidone |
| Drug Bank | DB02703 | Fusidic Acid |
| Drug Bank | DB01218 | Halofantrine |
| ClinicalTrials | NCT02057549 | Haloperidol |
| Drug Bank | DB08958 | Hexetidine |
| ClinicalTrials | NCT01054105 | Iloprost |
| ClinicalTrials | NCT02317159 | Imatinib |
| Drug Bank | DB08943 | Isoconazole |
| ClinicalTrials | NCT02457520 | Isotretinoin |
| Drug Bank | DB00602 | Ivermectin |
| ClinicalTrials | NCT00133627 | Ketotifen |
| ClinicalTrials | NCT01512953 | Lansoprazole |
| ClinicalTrials | NCT02703649 | Letrozole |
| Drug Bank | DB00589 | Lisuride |
| Drug Bank | DB01206 | Lomustine |
| Drug Bank | DB00836 | Loperamide |
| Drug Bank | DB00358 | Mefloquine |
| Drug Bank | DB00170 | Menadione |
| Drug Bank | DB00340 | Metixene |
| Drug Bank | DB00764 | Mometasone |
| Drug Bank | DB00600 | Monobenzone |
| ClinicalTrials | NCT02068404 | Nifedipine |
| Drug Bank | DB00336 | Nitrofural |
| Drug Bank | DB00540 | Nortriptyline |
| Drug Bank | DB00850 | Perphenazine |
| Drug Bank | DB01438 | Phenazopyridine |
| Drug Bank | DB00498 | Phenindione |
| Drug Bank | DB00925 | Phenoxybenzamine |
| ClinicalTrials | NCT02649413 | Prednisone |
| ClinicalTrials | NCT02364583 | Primaquine |
| Drug Bank | DB01168 | Procarbazine |
| Drug Bank | DB00433 | Prochlorperazine |
| Drug Bank | DB00387 | Procyclidine |
| Drug Bank | DB00420 | Promazine |
| Drug Bank | DB00344 | Protriptyline |
| Therapeutic Targets Database | DAP001419 | Quercetin |
| Drug Bank | DB00908 | Quinidine |
| ClinicalTrials | NCT01041092 | Raloxifene |
| Drug Bank | DB01301 | Rolitetracycline |
| ClinicalTrials | NCT01073007 | Simvastatin |
| Drug Bank | DB06820 | Sulconazole |
| Drug Bank | DB00605 | Sulindac |
| Therapeutic Targets Database | DAP001429 | Sulindac Sulfide |
| ClinicalTrials | NCT02496494 | Tacrolimus |
| ClinicalTrials | NCT00900744 | Tamoxifen |
| Therapeutic Targets Database | DAP000102 | Terfenadine |
| Drug Bank | DB00277 | Theophylline |
| Drug Bank | DB00679 | Thioridazine |
| Drug Bank | DB00208 | Ticlopidine |
| Drug Bank | DB01056 | Tocainide |
| Drug Bank | DB00755 | Tretinoin |
| Drug Bank | DB00831 | Trifluoperazine |
| Drug Bank | DB00432 | Trifluridine |
| Drug Bank | DB00726 | Trimipramine |
| Therapeutic Targets Database | DAP001337 | Troglitazone |
| Drug Bank | DB01624 | Zuclopenthixol |
