# Supplementary material for: Identification of Non-Electrophilic Nrf2 Activators from Approved Drugs
Source: Molecules. 2017 May 26;22(6):883. doi: 10.3390/molecules22060883 (PMC6152778; doi:10.3390/molecules22060883)

This file contains a part of concentration data and gel electrophoresis results of our RNA samples. Because the experiments were done in different batches, the information of earlier samples has been delated. We think that these data can prove the quantities and quality of our RNA samples.

No.	Sample Name	Concentration (μg/μL)	Vol. (μL)	A260/ A280	Result
1	DMSO 0-1	0.274	20	2.12	Qualified
2	DMSO 0-2	0.299	20	2.15	Qualified
3	DMSO 0-3	0.280	20	2.14	Qualified
4	DMSO 6-1	0.325	20	2.13	Qualified
5	DMSO 6-2	0.318	20	2.12	Qualified
6	DMSO 6-3	0.325	20	2.15	Qualified
7	DMSO 12-1	0.355	20	2.15	Qualified
8	DMSO 12-2	0.344	20	2.10	Qualified
9	DMSO 12-3	0.330	20	2.14	Qualified
10	DMSO 24-1	0.412	20	2.13	Qualified
11	DMSO 24-2	0.419	20	2.14	Qualified
12	DMSO 24-3	0.402	20	2.14	Qualified
13	Ast6-1	0.304	20	2.14	Qualified
14	Ast6-2	0.301	20	2.13	Qualified
15	Ast6-3	0.306	20	2.14	Qualified
16	Ast12-1	0.323	20	2.13	Qualified
17	Ast12-2	0.324	20	2.12	Qualified
18	Ast12-3	0.320	20	2.14	Qualified
19	Ast24-1	0.385	20	2.13	Qualified
20	Ast24-2	0.374	20	2.12	Qualified
21	Ast24-3	0.376	20	2.12	Qualified
22	Tamo t6-1	0.301	20	2.13	Qualified
23	Tamo 6-2	0.308	20	2.14	Qualified
24	Tamo 6-3	0.299	20	2.11	Qualified
25	Tamo12-1	0.320	20	2.14	Qualified
26	Tamo 12-2	0.319	20	2.13	Qualified
27	Tamo 12-3	0.326	20	2.14	Qualified
28	Tamo 24-1	0.375	20	2.10	Qualified
29	Tamo 24-2	0.384	20	2.13	Qualified
30	Tamo 24-3	0.390	20	2.11	Qualified

No.	Sample Name	Concentration (μg/μL)	Vol. (μL)	A260/ A280	Result
31	Chloro 0-1	0.282	20	2.11	Qualified
32	Chloro 0-2	0.289	20	2.13	Qualified
33	Chloro 0-3	0.292	20	2.12	Qualified
34	Chloro 6-1	0.314	20	2.13	Qualified
35	Chloro 6-2	0.317	20	2.13	Qualified
36	Chloro 6-3	0.315	20	2.13	Qualified
37	Chloro 12-1	0.345	20	2.14	Qualified
38	Chloro 12-2	0.356	20	2.12	Qualified
39	Chloro 12-3	0.332	20	2.14	Qualified
40	Chloro 24-1	0.413	20	2.12	Qualified
41	Chloro 24-2	0.408	20	2.13	Qualified
42	Chloro 24-3	0.414	20	2.14	Qualified
43	Trim6-1	0.310	20	2.12	Qualified
44	Trim6-2	0.307	20	2.13	Qualified
45	Trim6-3	0.303	20	2.15	Qualified
46	Trim12-1	0.325	20	2.13	Qualified
47	Trim12-2	0.330	20	2.12	Qualified
48	Trim12-3	0.323	20	2.11	Qualified
49	Trim24-1	0.377	20	2.13	Qualified
50	Trim24-2	0.386	20	2.12	Qualified
51	Trim24-3	0.386	20	2.13	Qualified
52	Diphe t6-1	0.299	20	2.13	Qualified
53	Diphe 6-2	0.301	20	2.14	Qualified
54	Diphe 6-3	0.307	20	2.13	Qualified
55	Diphe12-1	0.322	20	2.12	Qualified
56	Diphe 12-2	0.319	20	2.11	Qualified
57	Diphe 12-3	0.327	20	2.13	Qualified
58	Diphe 24-1	0.380	20	2.13	Qualified
59	Diphe 24-2	0.382	20	2.13	Qualified
60	Diphe 24-3	0.372	20	2.12	Qualified

No.	Sample Name	Concentration	Vol. (μL)	A260/ A280	Result
61	Dosu6-1	0.303	20	2.13	Qualified
62	Dosu6-2	0.309	20	2.12	Qualified
63	Dosu6-3	0.305	20	2.13	Qualified
64	Dosu12-1	0.319	20	2.12	Qualified
65	Dosu12-2	0.325	20	2.12	Qualified
66	Dosu12-3	0.325	20	2.14	Qualified
67	Dosu24-1	0.388	20	2.12	Qualified
68	Dosu24-2	0.383	20	2.11	Qualified
69	Dosu24-3	0.389	20	2.12	Qualified
70	Terfe6-1	0.305	20	2.14	Qualified
71	Terfe6-2	0.305	20	2.12	Qualified
72	Terfe6-3	0.310	20	2.13	Qualified
73	Terfe12-1	0.330	20	2.13	Qualified
74	Terfe12-2	0.327	20	2.13	Qualified
75	Terfe12-3	0.330	20	2.14	Qualified
76	Terfe24-1	0.388	20	2.15	Qualified
77	Terfe24-2	0.382	20	2.13	Qualified
78	Terfe24-3	0.389	20	2.14	Qualified
79	Hexe6-1	0.299	20	2.14	Qualified
80	Hexe6-2	0.304	20	2.11	Qualified
81	Hexe6-3	0.309	20	2.11	Qualified
82	Hexe12-1	0.328	20	2.11	Qualified
83	Hexe12-2	0.325	20	2.12	Qualified
84	Hexe12-3	0.334	20	2.12	Qualified
85	Hexe24-1	0.388	20	2.11	Qualified
86	Hexe24-2	0.384	20	2.14	Qualified
87	Hexe24-3	0.390	20	2.12	Qualified
88	Quini6-1	0.304	20	2.13	Qualified
89	Quini6-2	0.309	20	2.13	Qualified
90	Quini6-3	0.309	20	2.12	Qualified

No.	Sample Name	Concentration	Vol. (μL)	A260/ A280	Result
91	Quini12-1	0.322	20	2.11	Qualified
92	Quini12-2	0.329	20	2.12	Qualified
93	Quini12-3	0.322	20	2.12	Qualified
94	Quini24-1	0.378	20	2.11	Qualified
95	Quini24-2	0.384	20	2.12	Qualified
96	Quini24-3	0.382	20	2.13	Qualified
97	D,L-Sul6-1	0.302	20	2.13	Qualified
98	D,L-Sul6-2	0.304	20	2.12	Qualified
99	D,L-Sul6-3	0.298	20	2.12	Qualified
100	D,L-Sul12-1	0.322	20	2.12	Qualified
101	D,L-Sul12-2	0.320	20	2.11	Qualified
102	D,L-Sul12-3	0.318	20	2.14	Qualified
103	D,L-Sul24-1	0.376	20	2.14	Qualified
104	D,L-Sul24-2	0.368	20	2.13	Qualified
105	D,L-Sul24-3	0.382	20	2.14	Qualified

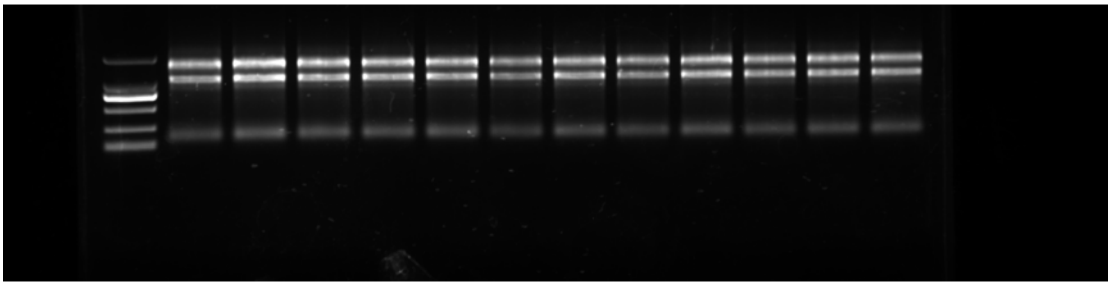

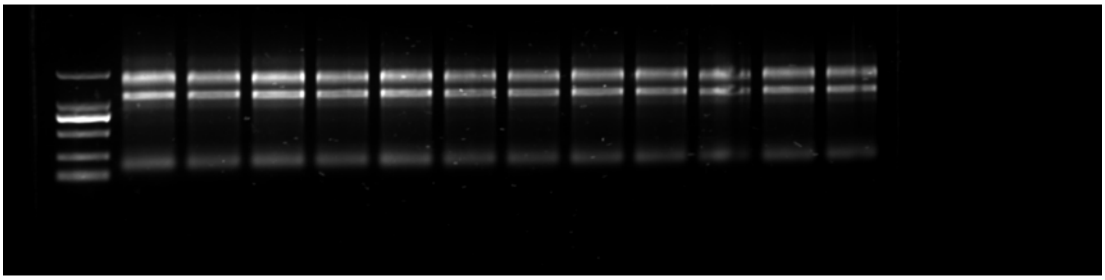

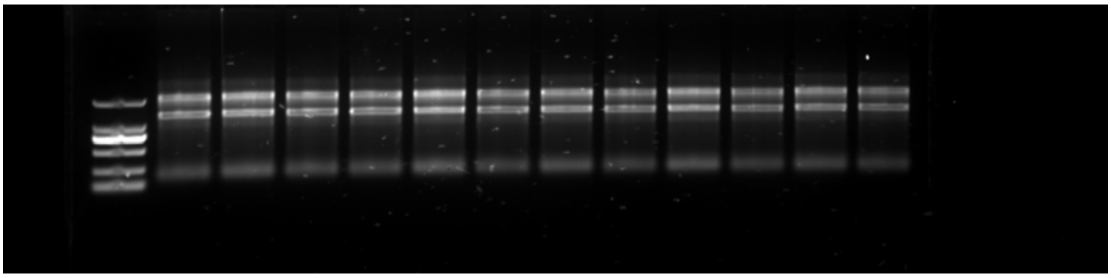

Supplement: Supplementary file 1 [file molecules-22-00883-s001.zip › RNA_samples_information.pdf]
