# Supplementary figures and images for: Identification of Non-Electrophilic Nrf2 Activators from Approved Drugs
Source: Molecules. 2017 May 26;22(6):883. doi: 10.3390/molecules22060883 (PMC6152778; doi:10.3390/molecules22060883)

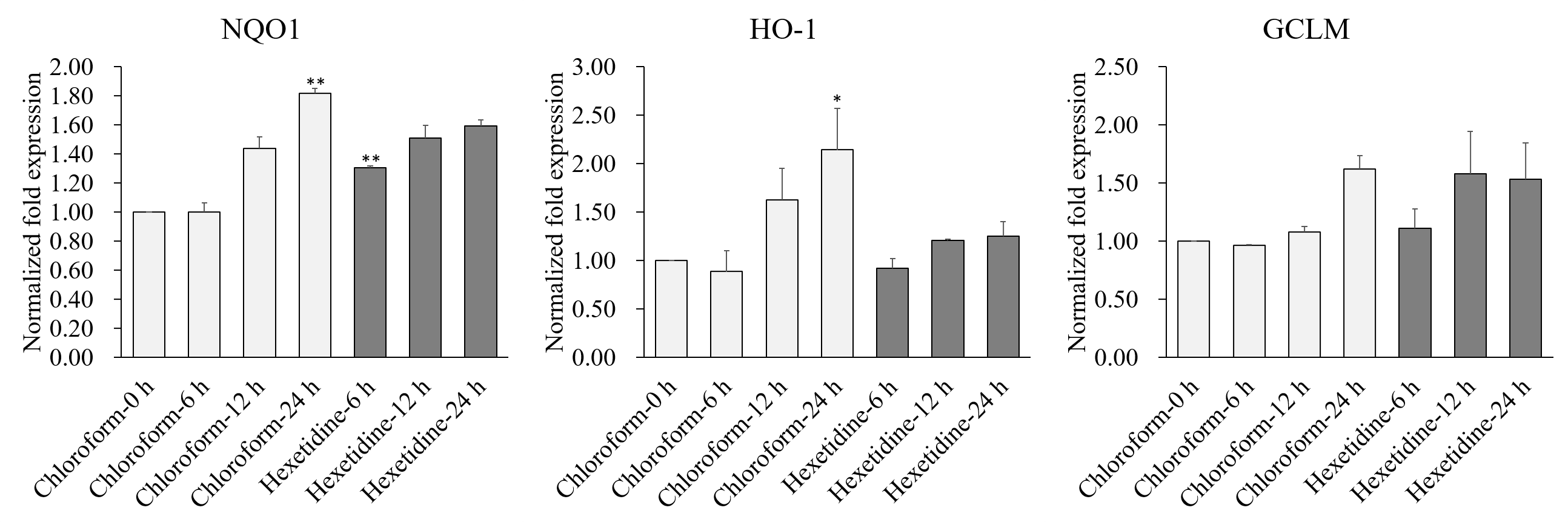

Supplement: Supplementary file 1 [file molecules-22-00883-s001.zip › Figure_S6.tif]

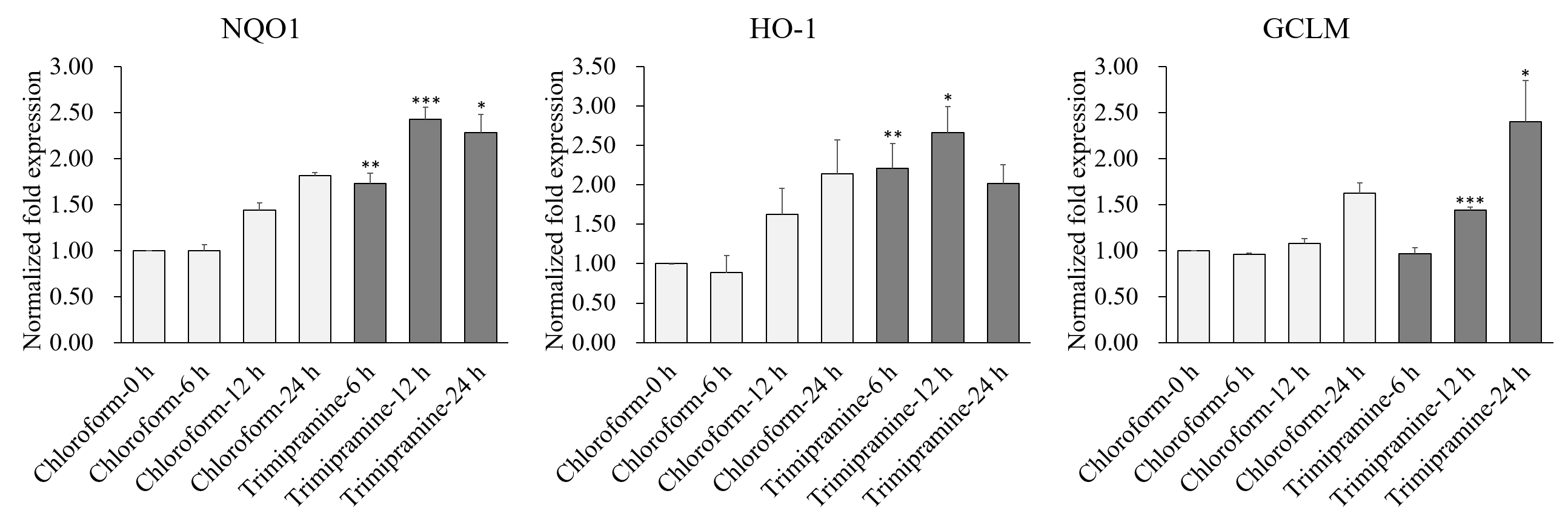

Supplement: Supplementary file 1 [file molecules-22-00883-s001.zip › Figure_S1.tif]

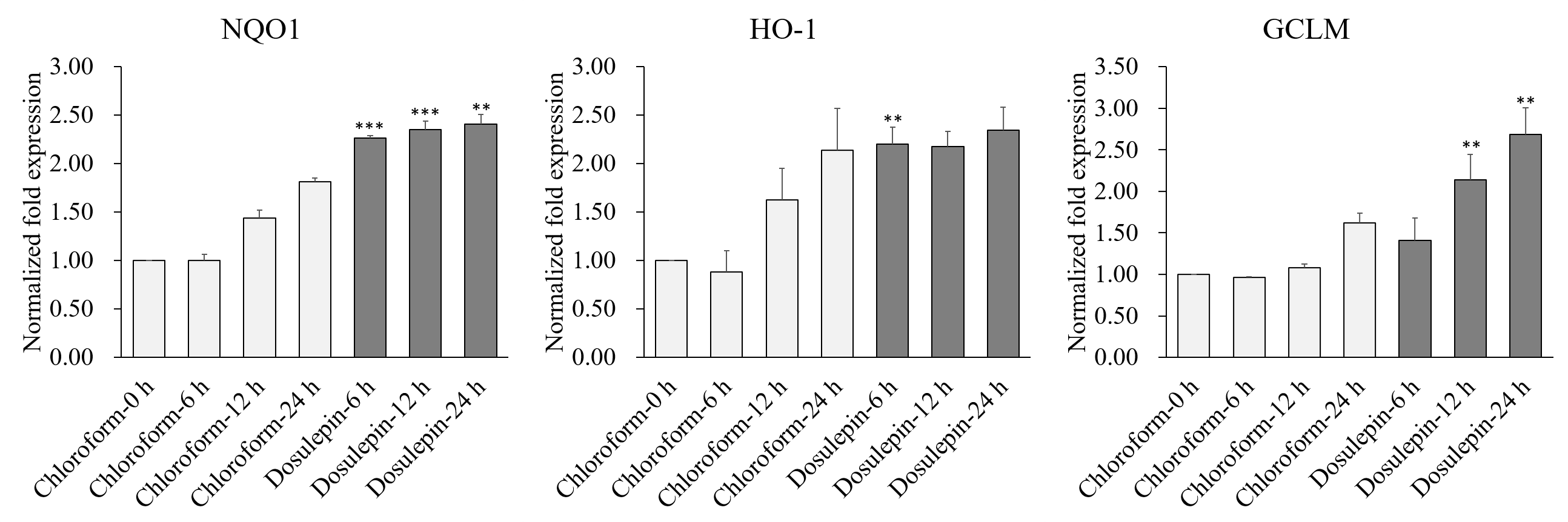

Supplement: Supplementary file 1 [file molecules-22-00883-s001.zip › Figure_S2.tif]

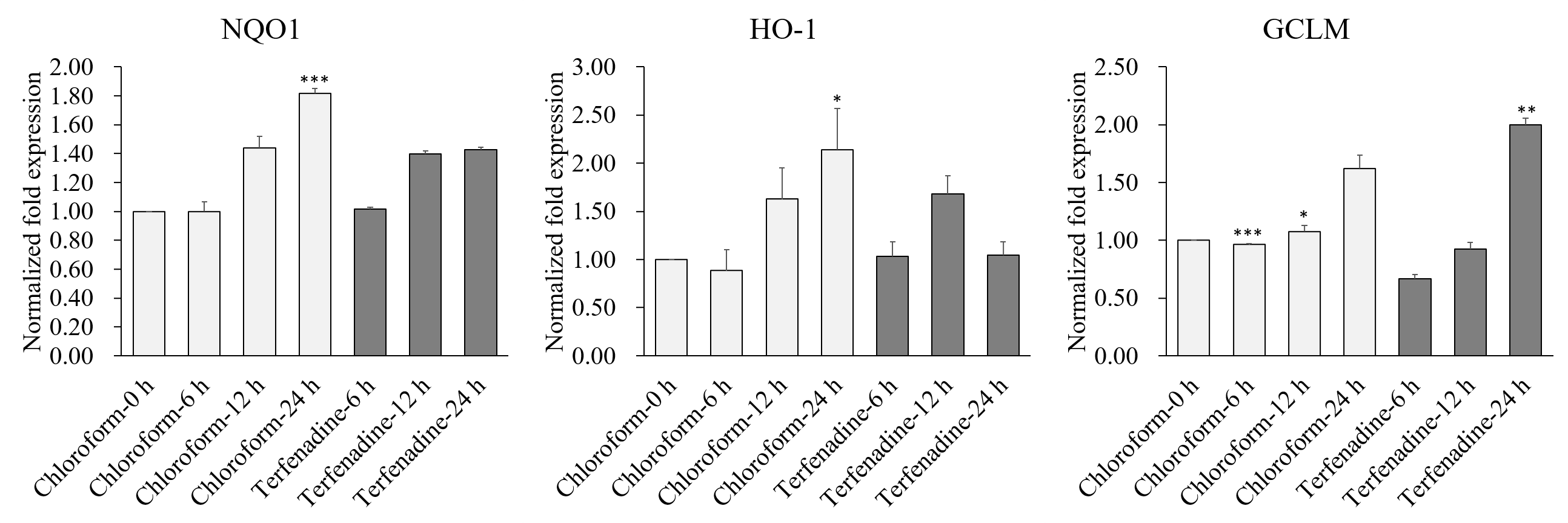

Supplement: Supplementary file 1 [file molecules-22-00883-s001.zip › Figure_S3.tif]

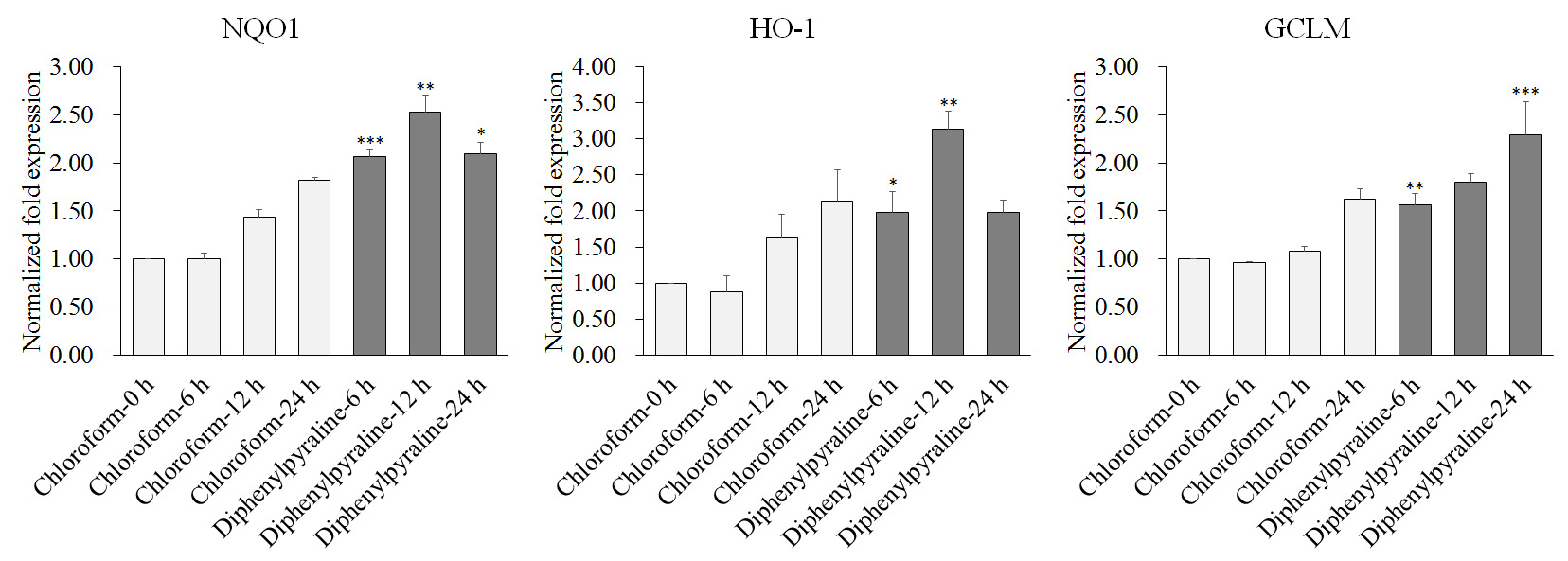

Supplement: Supplementary file 1 [file molecules-22-00883-s001.zip › Figure_S4.tif]

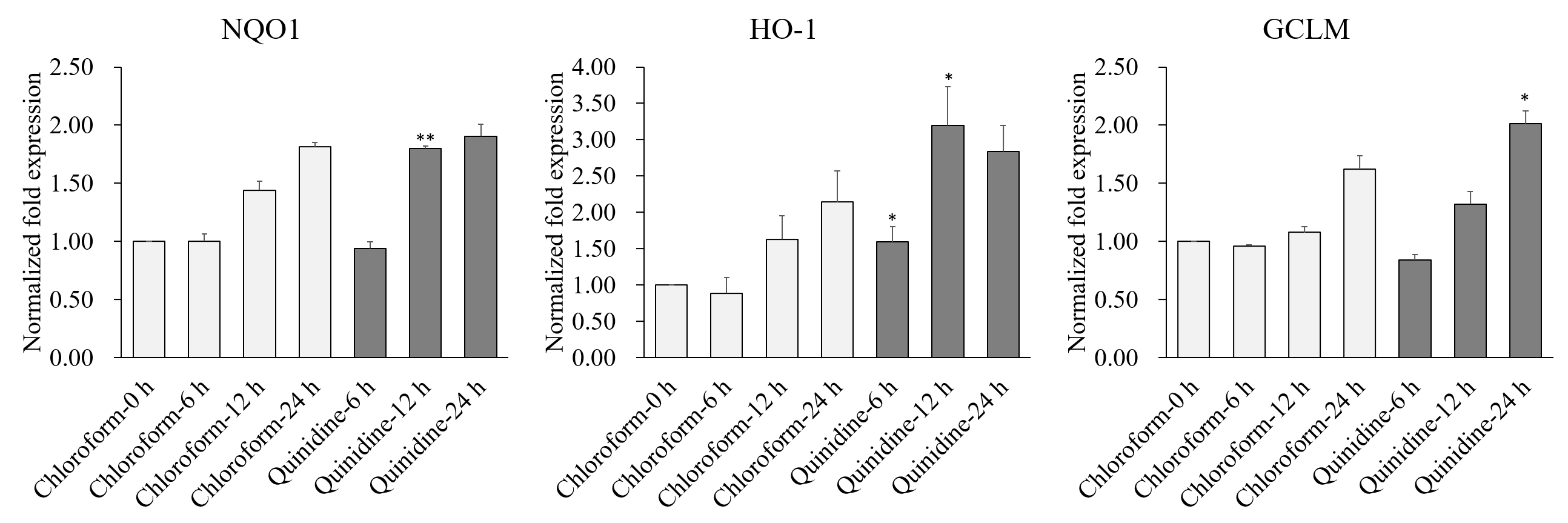

Supplement: Supplementary file 1 [file molecules-22-00883-s001.zip › Figure_S5.tif]
